# Interannual to decadal variability within and across the major Eastern Boundary Upwelling Systems

**DOI:** 10.1038/s41598-019-56514-8

**Published:** 2019-12-27

**Authors:** Giulia Bonino, Emanuele Di Lorenzo, Simona Masina, Doroteaciro Iovino

**Affiliations:** 1 Ocean Modeling and Data Assimilation Division, Centro Euro-Mediterraneo sui Cambiamenti Climatici, Bologna, Italy; 2 Università Ca’Foscari di Venezia, Venezia, Italy; 3 Program in Ocean Science & Engineering, Georgia Institute of Technology, Atlanta, USA

**Keywords:** Climate change, Climate sciences, Ocean sciences, Physical oceanography

## Abstract

Climate variability and climate change in Eastern Boundary Upwelling Systems (EBUS) affect global marine ecosystems services. We use passive tracers in a global ocean model hindcast at eddy-permitting resolution to diagnose EBUS low-frequency variability over 1958–2015 period. The results highlight the uniqueness of each EBUS in terms of drivers and climate variability. The wind forcing and the thermocline depth, which are potentially competitive or complementary upwelling drivers under climate change, control EBUS low-frequency variability with different contributions. Moreover, Atlantic and Pacific upwelling systems are independent. In the Pacific, the only coherent variability between California and Humboldt Systems is associated with El Ni*ñ*o Southern Oscillation. The remaining low-frequency variance is partially explained by the North and South Pacific expressions of the Meridional Modes. In the Atlantic, coherent variability between Canary and Benguela Systems is associated with upwelling trends, which are not dynamically linked and represent different processes. In the Canary, a negative upwelling trend is connected to the Atlantic Multi-decadal Oscillation, while in the Benguela, a positive upwelling trend is forced by a global sea level pressure trend, which is consistent with the climate response to anthropogenic forcing. The residual variability is forced by localized offshore high sea level pressure variability.

## Introduction

The Eastern Boundary Upwelling Systems (EBUS), such as the California Current System (CalCS), the Canary Current System (CanCS), the Humboldt Current System (HCS), and the Benguela Current System (BenCS), are among the most productive marine ecosystems, supplying up to 20% of the global fish catches, although they only cover approximately 1% of the total ocean^1–3^. Surface alongshore winds, together with the Coriolis effect, force the offshore water transport and the divergence of the surface flow, through Ekman Transport and Ekman suction, respectively, thereby lifting nutrient-rich deep waters into the euphotic layer. The nutrient-rich upwelled water, in addition to the sunlight, sustains the blooms of phytoplankton that are the foundation of the aquatic food web^2,4^. Recent studies have documented trends^3,5,6^ and decadal scale changes in the EBUS ecosystem structure^7,8^. Thus, understanding the low-frequency drivers and monitoring changes across EBUS is important. Bakun (1990) hypothesized an increase in upwelling-favourable winds (e.g. equatorward alongshore winds) due to the intensification of the continental-oceanic pressure gradient under global warming^9^. A more recent hypothesis suggests an alternative mechanism, whereby a poleward shift of the oceanic high-pressure system would stimulate latitude-dependent changes in the magnitude and timing of the upwelling winds^6,10^. Many studies demonstrate that the upwelling-favourable winds over EBUS intensify^2,3,9,11–13^ both in the past records and in the future projections although the driving mechanism is still debated, and conflicting results have been reported. Narayan *et al*. (2010) depicted decreasing trends for California^14^; Dewitte *et al*. (2012) and Tim *et al*. (2015) reported no significant trends in the Peru and Benguela systems^5,15^ and Pardo *et al*. (2011) and Sydeman *et al*. (2014a) showed a decreasing upwelling in the Canary systems, particularly along the Iberian coast^3,16^. As Garcia-Reyes *et al*. (2015) discussed in details in their review paper^2^, this lack of agreement about the future changes of the upwelling-favourable winds associated with the expected coastal warming makes any assessment of future of coastal temperatures and biogeochemistry challenging^3,17–25^. Coastal warming increases the water stratification and it can limit the effectiveness of upwelling to bring nutrient-rich deep waters up to the euphotic zone^2,26,27^. Increasing or decreasing of the upwelling-favourable winds can also mitigate or amplify the action of coastal warming. Therefore, the future changes in upwelling-favourable winds and stratification can be either complementary or competitive. Moreover, coastal trapped waves may also influence the water column stratification^28,29^, modulating coastal biogeochemical conditions^30^ and triggering vertical displacements of the thermocline^31^, which controls subsurface anomalies (e.g., salinity) and thus the impact on EBUS productivity^32,33^. The dataset used, the temporal coverage, and the upwelling index variable significantly influence the trend analysis results^2,3,34^.

From a broader climatic perspective, the evaluation of long-term trends from observations and model experiments is potentially complicated by climate modes, which likely exert some control on the upwelling low-frequency variability^2,4,13,34^. Many of the most significant upwelling modifications in EBUS - particularly in the Pacific Ocean - have been attributed to large-scale ocean-atmosphere processes^4^. Usually, ENSO is the leading mode of variability of the HCS^35^ and CalCS, and decadal oscillations like the Pacific Decadal Oscillation (PDO), and the North Pacific Gyre Oscillation (NPGO) are usually linked to California Current System variability^4,36,37^. In the Atlantic sector, however, the BenCS appears to not be affected by basin-scale thermocline perturbations (Atlantic El Ni*ñ*o), but highly influenced by small-scale local physical variability^38^. Nevertheless, Hagen *et al*. (2001) reported a potential influence of the Quasi-Biennial Oscillation (QBO)^39^, and the Antarctic Oscillation (AAO), and others (e.g., Dufois and Rouault (2012)) have suggested that the ENSO signal may be of great importance^40^. Several studies report a large influence of the North Atlantic Oscillation (NAO) and the Atlantic Multidecadal Oscillation (AMO) on upwelling magnitudes and interannual to decadal variability in the Canary/Iberian Current System^14,16,41^. In addition to the backdrop of these competing findings, there are no studies that report a interannual-to-decadal shared variability across all the EBUS, despite the well-known shared low-frequency variability between the northern and the southern Pacific Ocean, which is mainly due to ENSO.

In this context, the focus of this study is to understand the coherent and non-coherent low frequency variability across the EBUS, and to explore how it is linked to large-scale climate modes. The aims are to: (1) quantify the forcing dynamics (e.g., alongshore winds, wind stress curl, thermocline depth) that controls low-frequency modulations in each EBUS, (2) identify how the forcing is linked to large-scale climate dynamics, and finally (3) understand the extent to which large-scale climate dynamics imprint a coherent signal across EBUS. To conduct our analysis, we modelled ocean dynamics in upwelling areas using a global eddy-permitting configuration of the NEMO model^42^ from 1958 to 2015. In our simulation, the ocean in the EBUS domains is driven by a new wind product, obtained through a statistical downscaling and merging of the large-scale wind structures from JRA55do-v1.1^43^ with the high resolution QuikSCAT winds. In the ocean model, passive tracers were released at the subsurface (150–250 m) in order to identify a proxy for coastal upwelling strength. This approach enables the cumulative effects of the different upwelling drivers to be measured using a proxy of nutrient fluxes (e.g. the passive tracer), which integrates the upwelling variability (e.g. vertical ocean currents). In terms of understanding the low-frequency variability of productivity in upwelling systems, this approach is more effective with respect to the analyses of the individual forcing (e.g., alongshore winds, wind stress curl, etc). However, there are shortcomings associated with the changes over time in subsurface nutrient concentrations^38^, which can generate a low-frequency signal from the modulation of the upwelling source waters. Nevertheless, this approach does allow a direct quantification of the extent to which upwelling efficiency, which is linked to vertical velocities, can raise the deep parcel to the euphotic zone.

## Data and Methods

### Model configuration

The low-frequency variability of EBUS is investigated in a numerical study based on the state-of-the-art modelling system NEMO (version 3.6). This is a three-dimensional, free-surface, hydrostatic, primitive-equation global ocean general circulation model^44^ coupled to the Louvain-la-Neuve Sea Ice Model, LIM2^45^. Our configuration employs a global ORCA025 tripolar grid^42^ with $\frac{1^\circ }{4}$ horizontal resolution: ~27.75 km at the Equator, ~14  m at 60°N or 60°S. The vertical grid consists of 75 levels, spaced from 1 m near the surface to ~200 m at the bottom, with partial steps representing the bottom topography^46^. To study the EBUS variability, we performed an ocean-only simulation (hereafter TRD55) covering the period from 1958 to 2015, with initial conditions for December 1957 provided by December 2015 of an existing NEMO ORCA025 ocean simulation (i.e., same code and resolution as TRD55) forced by JRA55dov1.1 reanalysis^43^ (hereafter JRA55). The extreme sensitivity of EBUS to the precise structure of the wind, as reported in several studies^47–51^ encouraged us to develop a new high-resolution dataset of wind based on observations, which was then used to force TRD55. We computed a statistical downscaling of the JRA55 wind over EBUS using QuickSCAT wind retrievals, following the method developed and reported by Goubanova *et al*. (2011)^52^. The statistical relationship, which is required to perform the statistical downscaling, is based on a multilinear regression between the near-surface winds retrieved by the QuikSCAT scatterometer (predictand, gridded product from IFREMER: 0.25° × 0.25° spatial resolution, 1-day temporal resolution from 2000 to 2008) and the large-scale sea level pressure (SLP) and near-surface wind fields from JRA55 data (predictors) for the period where predictand is available, which is from 2000 to 2008. This statistical relation between predictand and predictors is then used to downscale the JRA55 surface winds throughout the entire 1958–2015 period, to obtain a new high resolution dataset (with the same resolution of predictand, 25 km), corrected by observations. This technique allows for the correction of coastal wind patterns off EBUS, due to the accuracy of QuikSCAT^53,54^. In JRA55, although significantly improving on ERAInterim^55^, the representation of wind drop off is still an open issue that can effect Ekman pumping, coastal upwelling^9^ and alongshore transport^56^. In addition, the resolution of the Japanese reanalysis (~55 km) is still too coarse to resolve upwelling dynamics, which are typically confined to within 30 km from the coast. The remaining turbulent variables (temperature and specific humidity at 2 m), the radiative fluxes and precipitation are provided by the JRA55dov1.1 reanalysis, as 3 h mean values.

### Passive tracer set up

**Figure 1:**
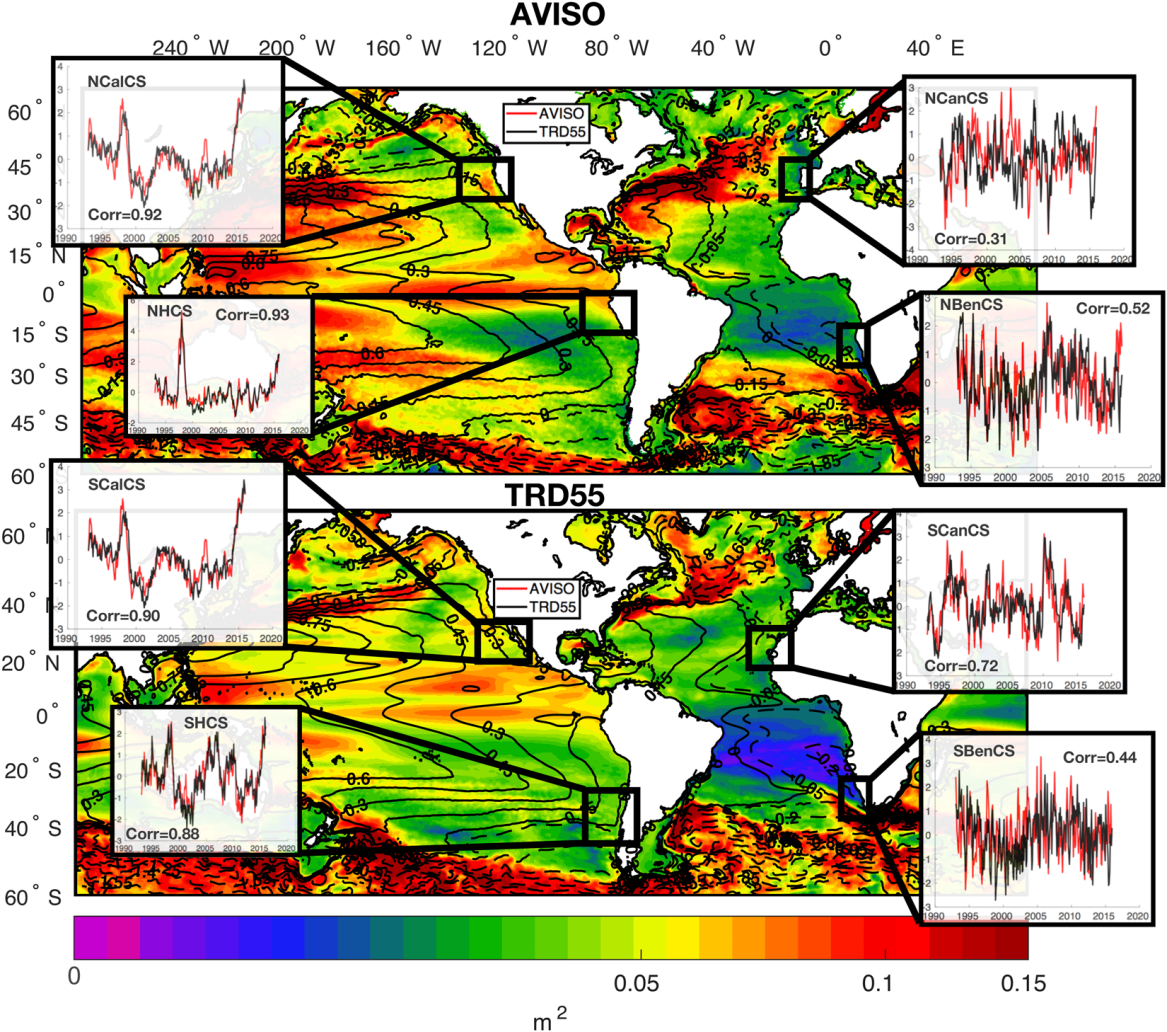
SSHa standard deviation (shaded areas) and mean SSHa (contour) for AVISO (top panel) and model solution (bottom panel) during 1993–2015 period; subplots: normalized SSHa from AVISO data (AVISO, red lines) and model solution (TRD55, black lines) during 1993–2015 period for each EBUS. Black squares indicate areas over which SSHa timeseries are calculated. “Corr” in the subplots indicate correlation coefficients between AVISO and TRD55 timeseries. This figure was plotted using MATLAB R2017a (https://www.mathworks.com/products/new_products/release2017a.html).

**Figure 2:**
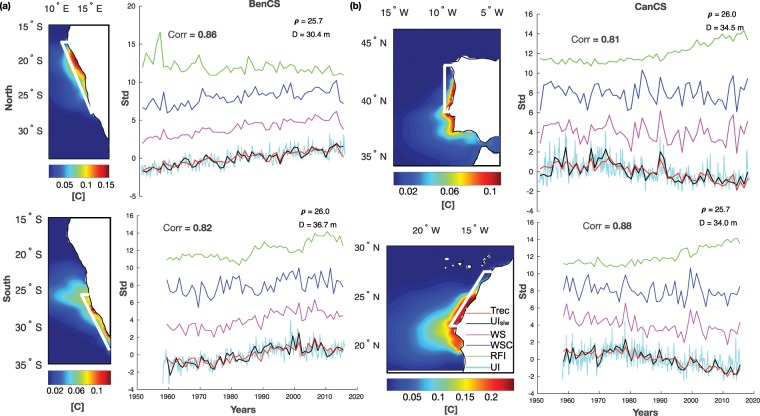
For (**a**) BenCS and (**b**) CanCS: Long-term mean (1958–2015) of surface passive tracers concentration released at subsurface at surface (left panels); low-frequency modulation of upwelling (*UI*_*slw*_, black line), alongshore wind stress (*WS*, purple line), wind stress curl (*WSC*, blue line) and *RFI* (*RFI*, green line) seasonal cycle and the reconstructed times series of upwelling (*Trec*, red line) (right panels). In cyan monthly timeseries of tracer anomaly (*UI*). Corr indicates the correlation between predictand and reconstructed timeseries; *ρ* indicates the isopycnal and *D* its mean depth. White boxes indicate the regions where the tracers have been released in the subsurface. *UI*_*slw*_, *WSC*, *WS*, *UI* time series are calculated at surface, while *RFI* is computed along the reference isopycnal *ρ*. All the times-series are normalized by their standard deviation. To distinguish time series they are stacked by a 4std vertical offset. This figure was plotted using MATLAB R2017a (https://www.mathworks.com/products/new_products/release2017a.html).

**Figure 3:**
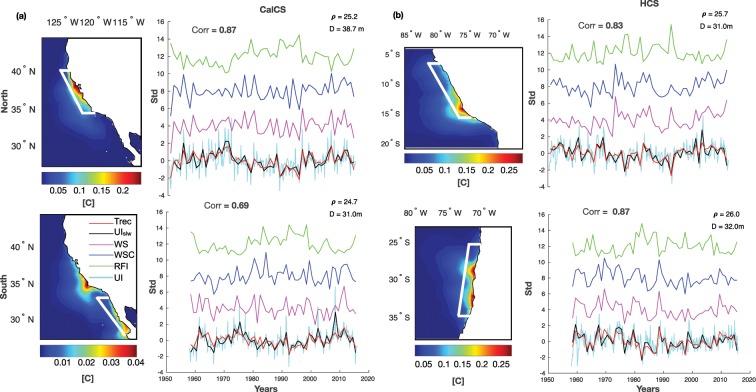
As Fig. 2 but for (**a**) CalCS and (**b**) HCS. This figure was plotted using MATLAB R2017a (https://www.mathworks.com/products/new_products/release2017a.html).

Here, we aim to identify the water masses originating at the subsurface (usually rich in nutrients) in each EBUS (see the black squares in Fig. 1) that can reach the surface through vertical advection. Thus, following the approach of Combes *et al*. (2013 and 2015)^57,58^, who investigated the upwelling and cross-shore transport variability in the California Current System and Humboldt Current System, we introduce passive tracers at subsurface in each EBUS. These are calculated in the model by a passive tracer advection-diffusion equation with a damping term:

1 $$\frac{\partial Tr}{\partial t}=-\,\overrightarrow{u}\cdot \nabla Tr+{A}_{H}{\nabla }_{H}^{2}Tr+\frac{\partial }{\partial z}({A}_{v}\frac{\partial Tr}{\partial z})+\tau (Tr-{T}_{0})$$

where *Tr* is the passive tracer concentration, *A*_*H*_ is the horizontal diffusivity, *A*_*V*_ is the vertical diffusivity, $\overrightarrow{u}$ is the velocity field, *T*_0_ is the damping term that controls the continuous source of tracer at sub-surface and *τ* is the damping timescale set to 6 months (required to avoid an infinite growth of passive tracer concentrations within the model domain). To address the upwelling of the subsurface water, we prescribed the damping term (*T*_0_) so that the passive tracer (*Tr*) is set to 1 in the coastal areas, as illustrated by the white rectangles in Figs. 2 and 3 (from the coast to 50 km offshore), and in the subsurface from 150 m to 250 m in depth.

EBUS provide a temporally and spatially heterogeneous environment^59^ and they are usually latitudinally divided in different upwelling regimes^38^, so we divide each EBUS into a northern and a southern part and we inject two independent tracers in each domain. We divide the Benguela System into two areas separated to the north of Lüderitz (at 26°S, see white square Figs. 2 and 3), hereinafter Northern Benguela (15°S–25°S, NBenCS) and Southern Benguela (26°S–34°S, SBenCS). The California domain is instead partitioned at Cape Mendocino (34°S) in the Northern California System (34°S–41°S, NCalCS) and Baja California (34°S-27°S, SCalCS). The subdivision is even more obvious for the Canary and Peru Systems, and in the former we inject the tracer over the Iberian Peninsula (40°N–44°N, WIP or NCanCS) and over the Moroccan sector (20°N–28°N, SCanCS), and in the latter along the Peruvian coast (5°S–15°S, NHCS) and along the Chilean coast (25°S–35°S, SHCS).

### Upwelling indices

In this work, we use the concentration of each subsurface tracer found at the surface as a measure of the upwelling efficiency. In particular, we define:

- The monthly upwelling efficiency index (*UI*): the monthly tracer concentration at the surface, from the coast to 150 km offshore and along the latitude band where the tracer is released (see white boxes in Figs. 2 and 3);
- The annual upwelling efficiency index (*UI*_*a*_): computed as *UI* but in annual mean;
- The low-frequency modulation of the upwelling efficiency seasonal cycle (*UI*_*slw*_): obtained by projecting the seasonal tracer concentration at the surface (from the coast to 50 km offshore and along the latitude where the tracer is released) onto the mean seasonal tracer spatial patterns. Upwelling intensity is considered in the selection of the season (see Supplementary Figure S1): in the Northern Hemisphere, May-June-July (MJJ) for NCalCS and SCalCS and April-May-June (AMJ) for SCanCS and SCanCS systems; in the Southern Hemisphere, upwelling December-January-February (DJF) in SBenCS, October-November-December (NDJ) along SHCS, and September-October-November (SON) in NBenCS and along NHCS.

Hereinafter, throughout the text and the figures, the term “upwelling” identifies the upwelling efficiency.

### Drivers indices

To investigate the drivers of upwelling, we examine wind forcing (alongshore wind stress and wind stress curl) and thermocline depth as forcing. Specifically:

- Alongshore wind stress index (*WS*): obtained by projecting the seasonal alongshore wind stress (from the coast to 50 km offshore and along the latitude where the tracer is released) onto the mean seasonal wind stress spatial patterns. Alongshore wind stress is calculated by rotating the u and v components of wind stress to the shoreline direction;
- Wind stress curl index (*WSC*): obtained by projecting the seasonal wind stress curl (from the coast to 150 km offshore and along the latitude where the tracer is released) onto the mean seasonal wind stress curl spatial patterns.
- Thermocline depth or Remote Forcing Index (*RFI*): the seasonal depth variation of a representative isopycnal below the mix layer depth (usually around 10 m) in time (see Figs. 2 and 3 for isopycnals definitions). RFI is a measure of the relative change of thermocline depth, which is also linked to stratification, and to the passage of coastal trapped waves. Therefore RFI is influenced by both the remote forcing (e.g., waves) and the local forcing (e.g., winds, surface heating). The thermocline depth and the stratification, which are lumped together in the RFI index, can modify upwelling differently: the former by modifying the availability of nutrients to upwelling, and the latter by modifying the source depth of upwelled waters. Moreover, the remote signal captured by the RFI index is influenced either by waves generated at the equator (e.g., generated during El Ni*ñ*o events^60^) or by those produced by localized wind events along the coastal current systems (e.g., along CalCS^61,62^). We first analysed the net heat flux (Qnet). Unlikely, due to the nature of these areas and the complex air-sea feedback, Qnet results to be intensified toward the ocean during upwelling^4^.

The time series obtained by the projection method capture temporal modulations of the mean field, and have a positive sign, even though for example the sign of the wind stress field is negative (e.g., NH systems). The seasons considered when computing the drivers indices are the same as those used to compute the *UI*_*slw*_ index.

### Gridded observational data

Sea Level Pressure (SLP) fields are obtained as monthly means from the National Centers for Environmental Prediction-National Center for Atmospheric Research reanalysis product^63^ and are provided on a 2.5° × 2.5° horizontal grid (hereinafter NCEP SLP). In addition, we use the SST data set from the National Climatic Data Center (NCDC): the National Oceanic and Atmospheric Administration Extended Reconstruction SST, version 3b, at 2° × 2° resolution product (hereinafter NOAA SST)^64^, which consists of monthly mean values from 1854 to the present. We restrict the period of record to 1958–2015 to match the model results. In addiction, for validation purposes we also use a 23-year time series of satellite altimetry, namely from 1993 to 2015, at $\frac{1^\circ }{3}\times \frac{1^\circ }{3}$ resolution provided by Collect Localisation Satellites (CLS, Toulose, France; hereinafter AVISO).

### Climate indices

The connection between upwelling variability and the large-scale climate is explored using correlation analyses with the following climate modes: the Multivariate ENSO Index (MEI^65^), the Pacific Meridional Mode (PMM^66^, the Atlantic Meridional Oscillation (AMO^67^), the South Pacific Meridional Mode (SPMM^68^) and the Tropical Pacific Decadal Variability (TPDV^69^) are investigated. The SPMM index is calculated from the data sets of NOAA SST anomalies (hereinafter NOAA SSTa) calculated with respect to 1958–2015 climatology. Following Zhang *et al*. (2014)^68^, the SPMM index is inferred by the regression of NOAA SSTa onto normalized NOAA SSTa time series averaged over the southeast Pacific (15°S–19°S, 103°W–107°W).

The significance of correlations is estimated based on the probability density function (PDF) of the cross-correlation coefficients betweenthe 2 time series y1 and y2. The PDF is built by computing the correlation of 5000 random pairs of time series that have the same autocorrelation of y1 and y2. The trend significance is evaluated based on the PDF of the trend coefficients.

## Results

### Model validation

We first use observed datasets to evaluate the performance of ocean hindcast TRD55 to simulate ocean variability. In Fig. 1, we compare modelled sea surface height anomaly (SSHa) standard deviation (shaded areas) and mean (contours) against the satellite altimetry AVISO SSHa data for the 1993–2015 period. In general, the standard deviation of the observed SSHa is slightly underestimated by TRD55 over BenCS, nevertheless, the amplitude and spatial structure of the model mean SSHa compares relatively well with AVISO. Maxima are located over the Indian Ocean and along the western boundaries of the ocean, while minima are over the Southern Ocean and along the eastern boundaries. The time series of the modelled SSHa variability along the coastal boundaries of each EBUS (subplots in Fig. 1), are also significantly correlated with the observations (black squares in Fig. 1). The modelled coastal SSHa explains a significant proportion of the sea level anomaly in all the EBUS. Major discrepancies are reported over the Atlantic basin. Off Benguela, and in particular off the Iberian coast, TDR55 exhibits some discrepancies from observations, likely reflecting the model’s inability to capture the internal variability induced by the Agulhas current in BenCS^38^ and by the Gibraltar Strait and the extension of the Gulf stream in the Western Iberian Peninsula^70^. In contrast, over the Pacific Ocean, the correlation between the modelled and observed variability is highly significant with R values between 0.88–0.93. For example, the strong positive anomalies during the warm ENSO events in 1998 and 2015 (El Nino years) are clearly evident in both the CalCS and HCS EBUS.

### Drivers and trends of low frequency modulation of upwelling efficiency

To assess the drivers of low-frequency upwelling variability, we first examine the low-frequency modulations of the upwelling seasonal cycle. We set up a linear regression model between the extracted seasonal timeseries of the tracer (*UI*_*slw*_) as predictand and the timesseries of forcing (e.g., alongshore wind stress, wind stress curl and RFI) as predictors (see the Data and Methods section for definitions of the indices). Figures 2 and 3 displays the regression results, while Table 1 reports the linear regression coefficients (*Coeff*) of each forcing and their contribution in percentage (%). Table 1 also displays the upwelling variance explained (*R*^2^) by each forcing. The coefficients (*Coeff*) from the multiple linear regression approach are identified using standard least square inversions, which are not sensitive to the order of the forcing term. The approach allows us to easily find the combination of forcing/weights that maximizes our ability to reconstruct the upwelling indices. In contrast, the upwelling variances explained (*R*^2^) by the forcing are identified as the square of the correlation between each forcing and the upwelling index. The reconstructed tracer signals (Trec, red lines), which are the time-series obtained as a result of the linear regression, are significantly correlated with *UI*_*slw*_, explaining about 70% of the variance in all the domains (see “Corr” correlation coefficient in each subplot). Thus, *UI*_*slw*_ is the result of low-frequency modulations of the forcing seasonal cycle, and high correlations of *UI*_*slw*_ with annual variation of tracers in all domains (e.g., *UI*_*a*_, third row of Table 1) indicate that the modulation of the upwelling seasonal cycle dominates the upwelling interannual variability. The low-frequency variability of the upwelling seasonal cycle in the Benguela systems is dominated by fluctuations in the wind forcing (Fig. 2a), accounting for 72% in the north and 66% in the south (Table 1, *R*^2^ column). The stratification and coastal-trapped waves are the major drivers in the other regions, modulating the intensity of the subsurface upwelled water at interannual timescale. In particular, as the negative correlation coefficients in Table 1 indicate, the deeper the isopycnal the weaker the upwelling intensity. RFI explains about 65% of the modelled upwelling modulation in HCS, about 70% in NCalCS and up to 73% in CanCs, while the wind forcing are minor contributors (see Table 1, *R*^2^ column). The simple regression model has significant ability to reconstruct the upwelling low-frequency variability. However, in the SCalCS the overall correlation (*R* = 0.69, Fig. 3a) and the variance explained by the forcing are lower (RFI *R*^2^ = 0.37, Table 1, *R*^2^ column). In fact, the residual variabilities not explained by the forcing, may be related to other mechanisms that could potentially impact upwelling efficiency. These, for example, are (1) long-term changes in the properties of upwelling source waters^33^, and (2) eddies transport and cross-shore geostrophic transport, which can suppress or enhance coastal upwelling^71,72^ and may mitigate future upwelling changes^73^. Horizontal advection and eddy activities are strong and unpredictable in this region^57,74^. Finally, the Benguela and Canary systems show significant trends: positive for Benguela and negative for Canary (Table 1, last row, and Fig. 2). In BenCS, the trends track changes in the wind (e.g., curl and alongshore components), while the trends seem to be driven by stratification changes in CanCS. Although historical records may not be indicative of climate change response, it is important to report that the positive upwelling trends in the BenCS may appear consistent with the findings by Bakun (1990), while there is not supportive evidences in the other regions, particularly with the negative trend in the CanCS (Fig. 2b). In addition, the trends coherency between the northern and the southern domains of Benguela system appears to not support either the theory on the poleward displacement of high pressure-systems, which should favour upwelling poleward^10^. Collectively, these results suggest that changes in upwelling are dominated by the interannual to multi-decadal variability of the forcing functions, which have different expressions in the different EBUS.

**Table 1:** Multi-linear regression coefficients (*Coeff*), contribution in percentage of predictors (WS, WSC, RFI) (%) and percentage of *UI* variance explained by the predictors (*R*^2^), from first to third rows.

NBenCS				SBenCS			NCanCS			SCanCS			NCalCS			SCalCS			NHCS			SHCS		
	Coeff	%	*R*^2^	Coeff	%	*R*^2^	Coeff	%	*R*^2^	Coeff	%	*R*^2^	Coeff	%	*R*^2^	Coeff	%	*R*^2^	Coeff	%	*R*^2^	Coeff	%	*R*^2^
**WS**	0.93	72.6	0.72	0.63	70.3	0.66	0.58	28.5	0.22	0.36	30.4	0.41	0.31	29.2	0.37	0.25	26.0	0.14	0.06	6.4	0.01	0.34	34.2	0.36
**WSC**	−0.18	14.5	0.43	0.09	11.0	0.31	0.30	20.2	0.06	0.15	11.8	0.14	−0.04	0.27	0.04	0.15	16.0	0.06	0.17	16.7	0.04	0.01	4.0	0.33
**RFI**	−0.16	12.8	0.24	0.17	18.8	0.35	−0.63	51.2	0.52	−0.69	57.8	0.73	−0.71	66.5	0.70	−0.57	58.1	0.37	−0.82	76.9	0.64	−0.62	61.8	0.65
*UI*_*slw*_–*U*_*Ia*_	0.94			0.78			0.91			0.88			0.94			0.95			0.87			0.79		
*UI*_*slw*_–**-Trend**	0.05*			0.05*			−0.04*			−0.04*			0.0004			0.01			−0.01			−0.01		

Correlation between low frequency modulation of upwelling seasonal cycle (*UI*_*slw*_) and long-term modulation of upwelling (e.g. annual mean, *UI*_*a*_). Last row reported upwelling index *UI*_*slw*_ trends in [std/year]. Stars (*) indicate significant values.

### Shared climate variability across EBUS

**Figure 4:**
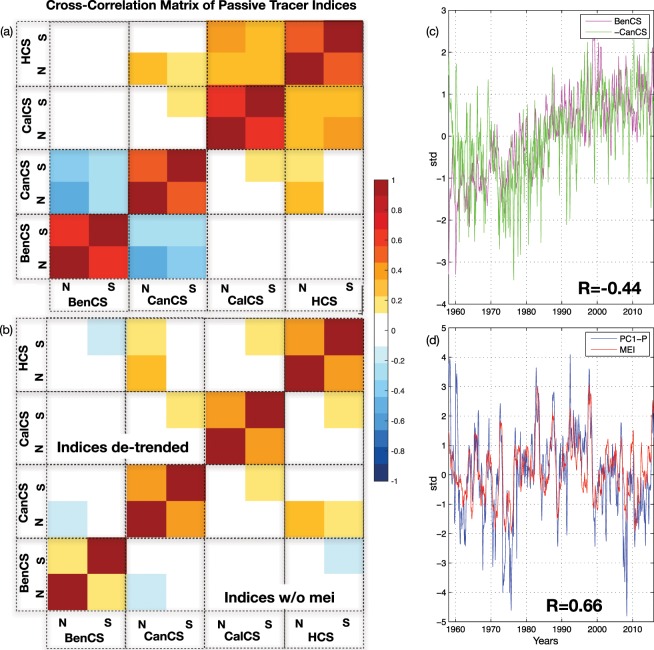
(**a**) Cross-correlation matrix of *UI*; (**b**) Cross-correlation matrix of detrended UI for CanCS and BenCS and UI without MEI signal for CanCS and HCS. N and S in (**a**,**b**) indicate the Northen and the Southern *UI* of each domain. (**c**) *UI* of BenCS and -CanCS timeseries. The BenCS and -CanCS timeseries are the sum of the northern and southern *UI*, (**d**) PC1 of Pacific *UI* with MEI index. *R* in (**c**,**d**) identifies correlation coefficients. The correlation coefficients are calculated considering the unchanged *UI* (e.g. CanCS not -CanCS). This figure was plotted using MATLAB R2017a (https://www.mathworks.com/products/new_products/release2017a.html).

**Figure 5:**
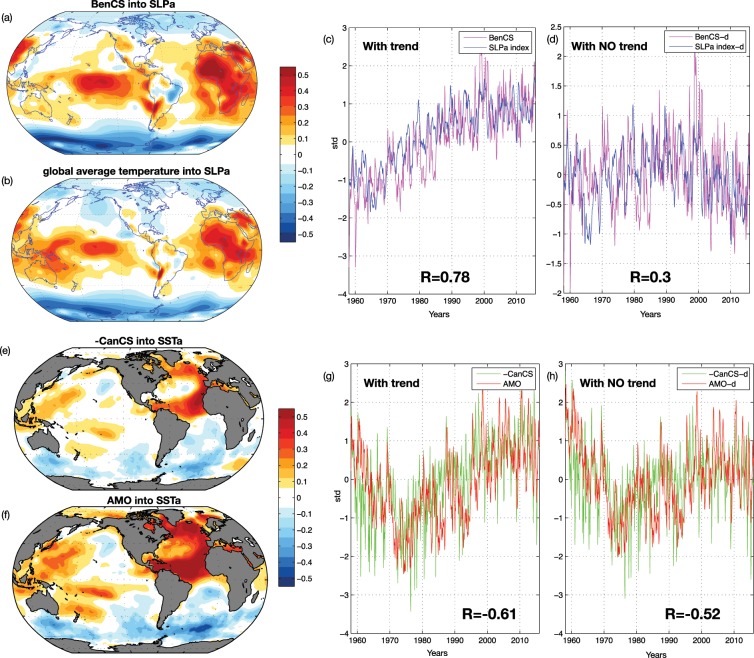
(**a**) Correlation pattern between BenCS *UI* and NCEP SLPa; (**b**) Correlation patterns between NOAA global average temperature (1958–2015) and NCEP SLPa; (c) BenCS UI and SLPa index; (**d**) detrended BenCS and detrended SLPa index. -d identifies detrended indices, R identifies significant correlation coefficients; (**e**) Correlation patterns between -CanCS UI and NOAA SSTa; (**f**) Correlation pattern between AMO index and NOAA SSTa; (**g**) -CanCS UI and AMO index; (**h**) detrended -CanCS *UI* and detrended AMO index. -d identifies detrended indices, R identifies significant correlation coefficients. The correlation coefficients are calculated considering the unchanged *UI* (e.g. CanCS not -CanCS). This figure was plotted using MATLAB R2017a (https://www.mathworks.com/products/new_products/release2017a.html).

**Figure 6:**
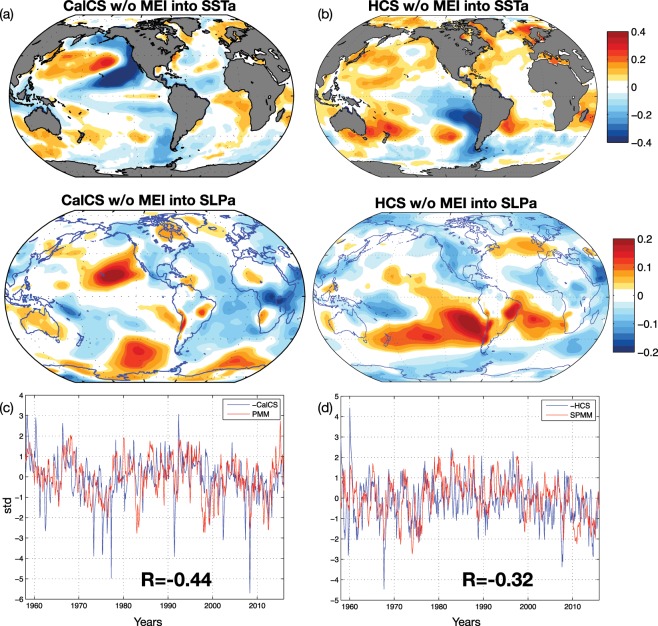
(**a**) Correlation patterns between CalCS UI without MEI signal and NOAA SSTa (top panel) and with NCEP SLPa (middle panel); (**b**) Correlation patterns between HCS UI without MEI signal and NOAA SSTa (top panel) and with NCEP SLPa (middle panel); (**c**) -CanCS UI without MEI signal and PMM index. (**d**) -HCS UI without MEI signal and SPMM index. R identifies significant correlation coefficients. The correlation coefficients are calculated considering the unchanged *UI *(e.g. HCS not -HCS). This figure was plotted using MATLAB R2017a (https://www.mathworks.com/products/new_products/release2017a.html).

**Figure 7:**
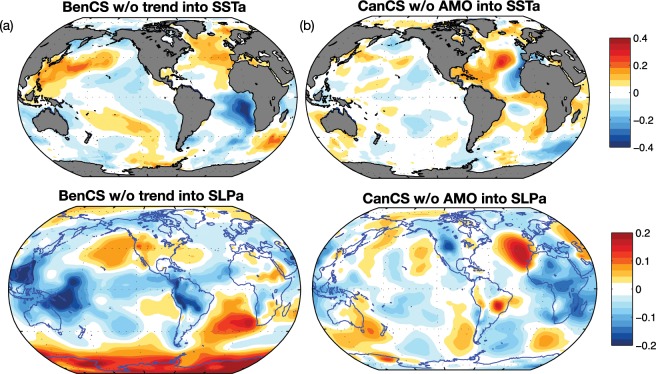
(**a**) Correlation patterns between BenCS UI without climate change signal (SLPa index) and NOAA SSTa (top panel) and NCEP SLPa (bottom panel); (**b**) Correlation patterns between CanCS UI without AMO index and NOAA SSTa (top panel) and NCEP SLPa (bottom panel). This figure was plotted using MATLAB R2017a (https://www.mathworks.com/products/new_products/release2017a.html).

The low-frequency variability of upwelling in each EBUS likely reflects the local expression of the large-scale modes of climate variability that modulates the local forcing functions (e.g., wind stress, curl, thermocline anomalies, etc.), and the response to local drivers that are not connected to large-scale climate variability. Here, we explore the relation between upwelling indices and large-scale climate dynamics to quantify the extent to which climate modes contribute to coherent signals across EBUS. To explore the coherent variability across EBUS, we first computed a cross-correlation matrix of the upwelling indices (Fig. 4a). This initial analysis reveals that the EBUS upwelling climate variability is in the first order not coherent between the Pacific and Atlantic basin. The upwelling systems correlate only with the systems located in the same basin, despite weak correlations between CalCS and CanCS. Specifically, the northern and the southern upwelling of each domain are coherent, BenCS and CanCS correlate negatively, and CalCS and HCS correlate positively with a maximum value of 0.5. The Atlantic systems anticorrelation is, as expected, linked to the trends (Fig. 4b), the negative upwelling trend in the Canary opposes the positive upwelling trend in the Benguela (hereinafter the UI are the sum of the northern and southern part, due to the coherency resulted in Fig. 4). In contrast, the Pacific basin coherent variability is connected to ENSO. The dominant mode of variability (PC1-P, see Figure S2 for the spatial pattern), obtained by performing EOF analyses of the UI of the Pacific, explains 50% of the California and Peru total variance with correlations of ~0.65 and ~0.6 with MEI and TPDV indices, respectively (Fig. 4d). The remaining low-frequency variance, after removing the trends from Atlantic systems and ENSO from the Pacific systems, reveals little coherence across EBUS (Fig. 4b). In the Atlantic, the trends in the EBUS appear to be linked to different processes. As discussed in Section 3.1, the BenCS trend is driven by wind changes while CanCS trends are sensitive to local (e.g., thermocline depth and stratification) and remote (e.g., coastal trapped waves) forcing. The spatial structures of the trends, inferred by correlating the BenCS with NCEP SLPa (Fig. 5a) and CanCS with NOAA SSTa (Fig. 5e), confirm their different nature. The BenCS pattern (Fig. 5a) resembles the climate change trend in global sea level pressure found in the reanalysis and in the observations by Gillet *et al*. (2013)^75^ (see their Fig. 1, top panels). To further confirm the role of climate change in the BenCS trend, we perform a correlation map between the global average temperature index (e.g. climate change proxy) from NOAA reanalyses with NCEP SLPa during the experiment period (Fig. 5b). The resulting map almost exactly tracks the trend structure in the SLPa (compare Fig. 5a,b). The high-pressure system over the African continent, characteristic of this pattern, leads to enhanced upwelling winds, consistent with the finding by Bakun (1990) of increased upwelling-favourable winds in a warming climate. Nevertheless, it is important to stress that the mechanism proposed by Bakun (1990), known as the Bakun Hypothesis, is not consistent with our results. The SLP climate trend does not show an intensification of the continental-oceanic pressure gradient^9^. The temporal relation between the SLPa climate change pattern and the BenCS upwelling was explored by correlating an index that tracks the temporal variability of the SLPa trend pattern with the BenCS UI. The SLPa index is obtained by projecting SLPa onto the Fig. 5b correlation pattern (e.g., the climate change signature in the global sea level pressure). The correlation analyses reveal that BenCS trends and variability (e.g., detrended signal) are both connected to the SLP trend pattern and its interannual to decadal modulations with correlations of ~0.78 and ~0.30 (Fig. 5c,d). An analysis of the CanCS trends spatial pattern in Fig. 5e, instead, reveals an AMO type pattern in both space and time (Fig. 5f). A correlation analysis with the climate mode confirms that CanCS trends and variability (e.g., a detrended signal) is significantly linked to the AMO with correlations of ~−0.61 and ~−0.52 (Fig. 5g,h). The AMO modulates the inter-hemispheric meridional gradient of SST and, in turn, of SLP over the Atlantic basin^67^. Thus, during its positive phase, atmospheric surface pressure differences between North and South Atlantic lead to a south-westerly wind anomaly in Northern Hemiphere^76–78^, which tends to weaken upwelling favourable winds along the Canary systems. In addition, during this phase, the warm SST over the north Atlantic enhances stratification, and thus weakens upwelling in the Canary systems. These variations of the surface forcing, induced by the global warming over BenCS and by AMO over CanCS, are consistent with trends and drivers described in Section 3.1. After removing the signals that lead to coherent variability in the EBUS of each basin through linear regressions, we examine the variability that is not connected to ENSO for CalCS and HCS, to the AMO for CanCS, and to the global climate change trend for BenCS. Upwelling indices of the residual low-frequency variability in each EBUS are then correlated with large-scale SSTa and SLPa to examine the spatial coherence of the upwelling drivers. We found that the residual upwelling variability is linked to atmospheric local forcing structures in both the Pacific and in the Atlantic, which are characterized by an offshore system of high sea level pressure in the offshore (Fig. 6 for CalCS and HCS, 7 for BenCS and CanCS). In the Pacific Ocean (Fig. 6), a correlation map of the *UI* residual indices with SSTa revealed structures that resemble the negative phase of the North and South Pacific Meridional Modes (see Chiang and Vimont (2004)^66^; Zhang *et al*.^68^), which are known to act independently^79,80^. This is not surprising given that the pressure systems driving the residual upwelling variability (Fig. 6, middle panels) project onto the excitation patterns of the Meridional Modes (MMs) by altering the strength of the off-equatorial trade winds. The modulation of the winds trigger the MMs over the Pacific through the wind-evaporation-SST feedback (Xie and Philander (1994)). In their negative phase, the MMs are characterized by upwelling favourable winds and strong cooling. The link to the MMs variability is further confirmed by correlating the North and South MM indices against the residual variability of the CalCS (~−0.44) and HCS (~−0.32) (Fig. 6c,d). Although the correlations pass the significance test for a 95% confidence level, there is an important proportion of non-shared variance between the MM indices and the UIs, which may results from the definition of the indices. However, the spatial structure in SSTa and SLPa are very consistent with the footprint of the MMs. For the Atlantic basin (Fig. 7), the residual variability inprint in the SLPa and SSTa is not associated with any known mode of climate variability. The local atmospheric modulation, characterized by the high sea level pressure in the offshore region (Fig. 7, bottom panels), is very localized for the CanCS and shows some related variability over the Antarctic for the BenCS. However, the projection of the BenCS onto the Antarctic SLPa does not appear connected to annular modes or other related Antarctic variability.

## Conclusions

In this work, we performed an ocean hindcast simulation for the period 1958 to 2015 using a global eddy-permitting ORCA025 configuration ($\frac{1^\circ }{4}$ of horizontal resolution) of the NEMO framework, with the aim of modelling and studying the interannual to decadal variability of the major Eastern Boundary Upwelling Systems. The numerical simulation is forced by the recent JRA55-dov.1.1 surface-atmospheric dataset (at 55 km of resolution) for driving ocean sea-ice models. Specifically, the winds from the large-scale wind structures of JRA55dov.1.1 have been statistically downscaled to 25 km of resolution using the high resolution QuikSCAT winds. To quantify the upwelling, we introduced an ensemble of passive tracers in the simulation, which are continuously released in the subsurface layer (150–250 m) in each EBUS over a region from the coast to 50 km offshore. The statistics of the concentration of these passive tracers at the surface (e.g. *UI*, *UI*_*slw*_ indices), which correspond to upwelled coastal water masses and ocean tracers (e.g., nutrients), enabling us to study the local and large-scale climate drivers of upwelling low-frequency variability and trends.

We first analysed the local drivers and trends of the low-frequency variability of the upwelling efficiency. We found that the common pattern favouring upwelling (e.g., equatorward wind stress, cyclonic wind stress curl and thermocline depth variation) explains the low frequency modulation of upwelling. The linear regression analysis showed that the wind forcing contribution to upwelling variability is dominant over the Benguela systems, while remote forcing of the thermocline and stratification forcing plays a crucial role in regulating upwelling fluctuations on the Canary, California and Humboldt systems (Figs. 2 and 3). The analysis also revealed upwelling trends in the Atlantic systems: the Benguela systems are characterized by positive trends, and the opposite occurs in the Canary systems. The Northern Canary upwelling negative trend is in line with previous studies (such as Pardo *et al*.^16^, Pérez *et al*.^81^ and Gómez-Gesteira *et al*.^82^), where negative trends are detected both in SST-based index and in wind stress. The interpretation of the Benguela positive trends are more complex, as the previous published results are conflicting. Depending on the period chosen for the analysis and the data set used, previous studies suggest no trend^20^, positive^14,41,83^ or negative trends^14,16,84^ for the upwelling in the Benguela systems. In the Pacific Ocean, our analysis showed no significant trends either in the upwelling and or in its drivers, in agreement with Combes *et al*.^57^, and Combes *et al*.^58^. Importantly, our results are in partially disagreement with the hypothesis of Bakun (1990) suggesting an increased upwelling due to increased wind stress induced by the intensification of the continental-oceanic pressure gradient under climate change conditions. Indeed, except over the Benguela systems, the wind stress forcing in the reanalysis product does not show significant positive trends. Furthermore, the wind trends coherency between the northern and southern domains of Benguela does not support either the hypothesis about the poleward displacement of high pressure-systems that should favour upwelling poleward^10^. Nevertheless, it should be mentioned that the presence or the absence of upwelling trends in the domains could be related to the dominance of the natural and internal variability in the historical records, but the likelihood of significant or non-significant greenhouse gas-related interactions cannot be completely dismissed. In summary, this statistical analysis on upwelling low-frequency variability suggests that the local (e.g., wind forcing, stratification and thermocline depth) and the remote (e.g., passage of coastal trapped waves) forcing, with different contribution in each EBUS, control the interannual upwelling variability. Thus, in order to predict and to propose hypotheses on the long-term variations in upwelling, identifying a proper index of upwelling in relation to the major drivers of the each domain is essential. In particular, both the coastal wind variations^9^ and the stratification have to be considered^4,26^ as potentially competitive or complementary drivers of upwelling variability under climate change (e.g., enhanced coastal temperature and stratification associated with stronger or weaker coastal winds). However, our analysis only considers the main mechanisms that drive the upwelling and no others possible processes that could impact the upwelling efficiency, such as the cross-shore transport via eddies and the geostrophic horizontal advection. Additional analyses, which include identifying other upwelling drivers, are the subjects of future work.

The second important issue addressed is the influence of the large-scale climate variability on long-term upwelling and the degree to which there is coherent low-frequency variability across EBUS. The variability associated with climate modes could be of importance to predict future perturbations at interannual to decadal time scales. Cross-correlation analyses among upwelling indices showed that the Atlantic and Pacific upwelling variabilities are mainly independent, while intra-basin domains variabilities present some coherency (Fig. 4). In the Pacific Ocean, the only coherent variability between the California and Humboldt systems is associated with the El Ni*ñ*o-Southern Oscillation (Fig. 4), while the remaining low-frequency variance is partially explained by the independent expressions of the North and South Pacific Meridional Modes (Fig. 6), which are characterized in their negative phases by strong cooling and alongshore wind stresses in the California and Humboldt systems. In contrast, in the Atlantic, the coherent variability between the Canary and Benguela systems is associated with trends in upwelling (Fig. 4). However, consistent with the previous analysis, these trends are not dynamically linked and represent different processes. In the Benguela, the positive upwelling trend is forced by the climate change trend in global sea level pressure^75^, which is characterized by a strong high-pressure system over the African continent that leads to enhanced upwelling winds. In the Canary systems, the negative trend in upwelling is closely connected with the low-frequency variability of the Atlantic Multi-decadal Oscillation, as previously suggested in the literature^14,16,41^(Fig. 5). Aside from the trend components, the Canary and Benguela systems showed no coherent variations and their residual variability is forced by local atmospheric variability, namely a system of high pressure in the offshore regions (Fig. 7). However, as García-Reyes *et al*.^2^ argued in their study, the extraction of long-term trends from observational records and model simulations over the Pacific could have been complicated by the influence of climate modes (e.g., ENSO). Thus, our results highlight the uniqueness of each EBUS in terms of drivers and climate variability. Signs of global warming, characterized by strong upwelling winds in a changing climate, are evident only over Benguela systems and, from a broader climate prospective, EBUS do not share variability, except from the well-known influence of ENSO on Pacific systems. The question of whether the variabilities observed here are indicative of interannual to multidecadal upwelling fluctuations is obscured by the length of the time series, by the variable used to evaluate upwelling and by the use of an ocean-only simulation. Extending the current analysis to a longer period, with coupled models and with the same passive tracers approach, will help to clarify these issues, enabling the results to be compared, and to confirm any unexpected teleconnections between upwelling systems.

## Supplementary information

Supplementary Information

## Data Availability

The datasets generated during and/or analysed during the current study are available from the corresponding author on reasonable request.

## References

[1] Pauly D, Christensen V (1995). Primary production required to sustain global fisheries. Nature.

[2] Garca-Reyes M (2015). Under pressure: Climate change, upwelling, and eastern boundary upwelling ecosystems. Frontiers in Marine Science.

[3] Sydeman WJ (2014). Climate change and wind intensification in coastal upwelling ecosystems. Science.

[4] Jacox MG, Bograd SJ, Hazen EL, Fiechter J (2015). Sensitivity of the california current nutrient supply to wind, heat, and remote ocean forcing. Geophysical Research Letters.

[5] Tim N, Zorita E, Hünicke B, Yi X, Emeis K-C (2016). The importance of external climate forcing for the variability and trends of coastal upwelling in past and future climate. Ocean Science.

[6] Wang D, Gouhier TC, Menge BA, Ganguly AR (2015). Intensification and spatial homogenization of coastal upwelling under climate change. Nature.

[7] Lehodey P (2006). Climate variability, fish, and fisheries. Journal of Climate.

[8] Parrish R, Schwing FB, Mendelssohn R (2000). Mid-latitude wind stress: the energy source for climatic shifts in the north pacific ocean. Fish. Oceanogr..

[9] Bakun A (1990). Global climate change and intensification of coastal ocean upwelling. Science.

[10] Rykaczewski RR (2015). Poleward displacement of coastal upwelling-favorable winds in the ocean’s eastern boundary currents through the 21st century. Geophysical Research Letters.

[11] Xiu P, Chai F, Curchitser EN, Castruccio FS (2018). Future changes in coastal upwelling ecosystems with global warming: The case of the california current system. Scientific reports.

[12] Garreaud RD, Falvey M (2009). The coastal winds off western subtropical south america in future climate scenarios. International Journal of Climatology: A Journal of the Royal Meteorological Society.

[13] Bakun A, Field DB, Redondo-Rodriguez A, Weeks SJ (2010). Greenhouse gas, upwelling-favorable winds, and the future of coastal ocean upwelling ecosystems. Global Change Biology.

[14] Narayan N, Paul A, Mulitza S, Schulz M (2010). Trends in coastal upwelling intensity during the late 20th century. Ocean Science.

[15] Dewitte B (2012). Change in el nino flavours over 1958–2008: Implications for the long-term trend of the upwelling off peru. Deep Sea Research Part II: Topical Studies in Oceanography.

[16] Pardo PC, Padn XA, Gilcoto M, Farina-Busto L, Pérez FF (2011). Evolution of upwelling systems coupled to the long-term variability in sea surface temperature and ekman transport. Climate Research.

[17] Mendelssohn R, Schwing F (2002). Common and uncommon trends in sst and wind stress in the california and peru–chile current systems. Progress in Oceanography.

[18] Arstegui J (2009). Sub-regional ecosystem variability in the canary current upwelling. Progress in Oceanography.

[19] Belkin IM (2009). Rapid warming of large marine ecosystems. Progress in Oceanography.

[20] Demarcq H (2009). Trends in primary production, sea surface temperature and wind in upwelling systems (1998–2007). Progress in Oceanography.

[21] Lebassi B (2009). Observed 1970–2005 cooling of summer daytime temperatures in coastal california. Journal of Climate.

[22] Garca-Reyes, M. & Largier, J. Observations of increased wind-driven coastal upwelling off central california. *Journal of Geophysical Research: Oceans* **115** (2010).

[23] Rouault M, Pohl B, Penven P (2010). Coastal oceanic climate change and variability from 1982 to 2009 around south africa. African Journal of Marine Science.

[24] Gutiérrez Dimitri, Bouloubassi Ioanna, Sifeddine Abdelfettah, Purca Sara, Goubanova Katerina, Graco Michelle, Field David, Méjanelle Laurence, Velazco Federico, Lorre Anne, Salvatteci Renato, Quispe Daniel, Vargas Gabriel, Dewitte Boris, Ortlieb Luc (2011). Coastal cooling and increased productivity in the main upwelling zone off Peru since the mid-twentieth century. Geophysical Research Letters.

[25] Salvanes AGV (2015). Spatial dynamics of the bearded goby and its key fish predators off n amibia vary with climate and oxygen availability. Fisheries oceanography.

[26] Di Lorenzo E, Miller AJ, Schneider N, McWilliams JC (2005). The warming of the california current system: Dynamics and ecosystem implications. Journal of Physical Oceanography.

[27] Brady RX, Lovenduski NS, Alexander MA, Jacox M, Gruber N (2019). On the role of climate modes in modulating the air–sea co 2 fluxes in eastern boundary upwelling systems. Biogeosciences.

[28] Pietri A (2014). Impact of a coastal-trapped wave on the near-coastal circulation of the peru upwelling system from glider data. Journal of Geophysical Research: Oceans.

[29] Bachèlery M-L, Illig S, Dadou I (2016). Interannual variability in the s outh-e ast a tlantic o cean, focusing on the b enguela u pwelling s ystem: Remote versus local forcing. Journal of Geophysical Research: Oceans.

[30] Echevin V (2014). Intraseasonal variability of nearshore productivity in the northern humboldt current system: The role of coastal trapped waves. Continental Shelf Research.

[31] Pizarro, O., Shaffer, G., Dewitte, B. & Ramos, M. Dynamics of seasonal and interannual variability of the peru-chile undercurrent. *Geophysical Research Letters* **29** (2002).

[32] Pozo Buil M, Di Lorenzo E (2017). Decadal dynamics and predictability of oxygen and subsurface tracers in the california current system. Geophysical Research Letters.

[33] Rykaczewski, R. R. & Dunne, J. P. Enhanced nutrient supply to the california current ecosystem with global warming and increased stratification in an earth system model. *Geophysical Research Letters* **37** (2010).

[34] Tim N, Zorita E, Hünicke B (2015). Decadal variability and trends of the benguela upwelling system as simulated in a high-resolution ocean simulation. Ocean Science.

[35] Minobe S, Mantua N (1999). Interdecadal modulation of interannual atmospheric and oceanic variability over the north pacific. Progress in Oceanography.

[36] Chhak, K. & Di Lorenzo, E. Decadal variations in the california current upwelling cells. *Geophysical Research Letters* **34** (2007).

[37] Di Lorenzo, E. *et al*. North pacific gyre oscillation links ocean climate and ecosystem change. *Geophysical Research Letters* **35** (2008).

[38] Chavez FP, Messié M (2009). A comparison of eastern boundary upwelling ecosystems. Progress in Oceanography.

[39] Hagen E, Feistel R, Agenbag JJ, Ohde T (2001). Seasonal and interannual changes in intense benguela upwelling (1982–1999). Oceanologica Acta.

[40] Dufois F, Rouault M (2012). Sea surface temperature in false bay (south africa): towards a better understanding of its seasonal and inter-annual variability. Continental Shelf Research.

[41] Cropper TE, Hanna E, Bigg GR (2014). Spatial and temporal seasonal trends in coastal upwelling off northwest africa, 1981–2012. Deep Sea Research Part I: Oceanographic Research Papers.

[42] Madec G, Imbard M (1996). A global ocean mesh to overcome the north pole singularity. Climate Dynamics.

[43] Tsujino Hiroyuki, Urakawa Shogo, Nakano Hideyuki, Small R. Justin, Kim Who M., Yeager Stephen G., Danabasoglu Gokhan, Suzuki Tatsuo, Bamber Jonathan L., Bentsen Mats, Böning Claus W., Bozec Alexandra, Chassignet Eric P., Curchitser Enrique, Boeira Dias Fabio, Durack Paul J., Griffies Stephen M., Harada Yayoi, Ilicak Mehmet, Josey Simon A., Kobayashi Chiaki, Kobayashi Shinya, Komuro Yoshiki, Large William G., Le Sommer Julien, Marsland Simon J., Masina Simona, Scheinert Markus, Tomita Hiroyuki, Valdivieso Maria, Yamazaki Dai (2018). JRA-55 based surface dataset for driving ocean–sea-ice models (JRA55-do). Ocean Modelling.

[44] Madec, G. *NEMO ocean engine* (2015).

[45] Fichefet T, Maqueda MM (1997). Sensitivity of a global sea ice model to the treatment of ice thermodynamics and dynamics. Journal of Geophysical Research: Oceans.

[46] Bernard B (2006). Impact of partial steps and momentum advection schemes in a global ocean circulation model at eddy-permitting resolution. Ocean dynamics.

[47] Desbiolles F, Blanke B, Bentamy A (2014). Short-term upwelling events at the western african coast related to synoptic atmospheric structures as derived from satellite observations. Journal of Geophysical Research: Oceans.

[48] Capet XJ, Marchesiello P, McWilliams JC (2004). Upwelling response to coastal wind profiles. Geophysical Research Letters.

[49] Small RJ, Curchitser E, Hedstrom K, Kauffman B, Large WG (2015). The benguela upwelling system: Quantifying the sensitivity to resolution and coastal wind representation in a global climate model. Journal of Climate.

[50] Pickett, M. H. & Paduan, J. D. Ekman transport and pumping in the california current based on the us navy high-resolution atmospheric model (coamps). *Journal of Geophysical Research: Oceans* **108** (2003).

[51] Echevin V, Goubanova K, Belmadani A, Dewitte B (2012). Sensitivity of the humboldt current system to global warming: a downscaling experiment of the ipsl-cm4 model. Climate Dynamics.

[52] Goubanova K (2011). Statistical downscaling of sea-surface wind over the peru-chile upwelling region: diagnosing the impact of climate change from the ipsl-cm4 model. Climate Dynamics.

[53] Garreaud R, Muñoz RC (2005). The low-level jet off the west coast of subtropical south america: Structure and variability. Monthly Weather Review.

[54] Dewitte, B. *et al*. Modes of covariability between sea surface temperature and wind stress intraseasonal anomalies along the coast of peru from satellite observations (2000–2008). *Journal of Geophysical Research: Oceans* **116** (2011).

[55] Bonino G, Masina S, Iovino D, Storto A, Tsujino H (2019). Eastern boundary upwelling systems response to different atmospheric forcing in a global eddy-permitting ocean model. Journal of Marine Systems.

[56] Marchesiello P, McWilliams JC, Shchepetkin A (2003). Equilibrium structure and dynamics of the california current system. Journal of physical Oceanography.

[57] Combes V (2013). Cross-shore transport variability in the california current: Ekman upwelling vs. eddy dynamics. Progress in Oceanography.

[58] Combes V, Hormazabal S, Di Lorenzo E (2015). Interannual variability of the subsurface eddy field in the southeast pacific. Journal of Geophysical Research: Oceans.

[59] Wooster WS (1963). Eastern boundary currents. The sea.

[60] Frischknecht M, Münnich M, Gruber N (2015). Remote versus local influence of enso on the c alifornia current system. Journal of Geophysical Research: Oceans.

[61] Chapman DC (1987). Application of wind-forced, long, coastal-trapped wave theory along the california coast. Journal of Geophysical Research: Oceans.

[62] Connolly TP, Hickey BM, Shulman I, Thomson RE (2014). Coastal trapped waves, alongshore pressure gradients, and the california undercurrent. Journal of Physical Oceanography.

[63] Kalnay E (1996). The ncep/ncar 40-year reanalysis project. Bulletin of the American meteorological Society.

[64] Smith TM, Reynolds RW (2005). A global merged land–air–sea surface temperature reconstruction based on historical observations (1880–1997). Journal of Climate.

[65] Wolter K, Timlin MS (2011). El niño/southern oscillation behaviour since 1871 as diagnosed in an extended multivariate enso index (mei. ext). International Journal of Climatology.

[66] Chiang JC, Vimont DJ (2004). Analogous pacific and atlantic meridional modes of tropical atmosphere–ocean variability. Journal of Climate.

[67] Enfield DB, Mestas-Nuñez AM, Trimble PJ (2001). The atlantic multidecadal oscillation and its relation to rainfall and river flows in the continental us. Geophysical Research Letters.

[68] Zhang H, Clement A, Di Nezio P (2014). The south pacific meridional mode: A mechanism for enso-like variability. Journal of Climate.

[69] Choi J, An S-I (2013). Quantifying the residual effects of enso on low-frequency variability in the tropical pacific. International Journal of Climatology.

[70] Palter JB (2015). The role of the gulf stream in european climate. Annual review of marine science.

[71] Marchesiello P, Estrade P (2010). Upwelling limitation by onshore geostrophic flow. Journal of Marine Research.

[72] Jacox MG, Edwards CA, Hazen EL, Bograd SJ (2018). Coastal upwelling revisited: Ekman, bakun, and improved upwelling indices for the us west coast. Journal of Geophysical Research: Oceans.

[73] Oerder V (2015). Peru-chile upwelling dynamics under climate change. Journal of Geophysical Research: Oceans.

[74] Cessi P, Wolfe CL (2009). Eddy-driven buoyancy gradients on eastern boundaries and their role in the thermocline. Journal of Physical Oceanography.

[75] Gillett NP, Fyfe JC, Parker DE (2013). Attribution of observed sea level pressure trends to greenhouse gas, aerosol, and ozone changes. Geophysical Research Letters.

[76] Wang C, Dong S, Evan AT, Foltz GR, Lee S-K (2012). Multidecadal covariability of north atlantic sea surface temperature, african dust, sahel rainfall, and atlantic hurricanes. Journal of Climate.

[77] Gill A (1980). Some simple solutions for heat-induced tropical circulation. Quarterly Journal of the Royal Meteorological Society.

[78] Green B, Marshall J, Donohoe A (2017). Twentieth century correlations between extratropical sst variability and itcz shifts. Geophysical Research Letters.

[79] Ding R, Li J, Tseng Y-H (2015). The impact of south pacific extratropical forcing on enso and comparisons with the north pacific. Climate Dynamics.

[80] Ding R, Li J, Tseng Y-H, Sun C, Xie F (2017). Joint impact of north and south pacific extratropical atmospheric variability on the onset of enso events. Journal of Geophysical Research: Atmospheres.

[81] Pérez FF (2010). Plankton response to weakening of the iberian coastal upwelling. Global Change Biology.

[82] Gómez-Gesteira M (2011). The state of climate in nw iberia. Climate Research.

[83] Santos F (2012). Differences in coastal and oceanic sst trends due to the strengthening of coastal upwelling along the benguela current system. Continental Shelf Research.

[84] Gómez-Gesteira M (2008). Spatio-temporal upwelling trends along the canary upwelling system (1967–2006). Annals of the New York Academy of Sciences.

