# Supplementary material for: Interannual to decadal variability within and across the major Eastern Boundary Upwelling Systems
Source: Sci Rep. 2019 Dec 27;9:19949. doi: 10.1038/s41598-019-56514-8 (PMC6934820; doi:10.1038/s41598-019-56514-8)
Supplement: Supplementary file 1 — Supplementary Information [file 41598_2019_56514_MOESM1_ESM.pdf]

Interannual to decadal variability within and across the major Eastern Boundary Upwelling Systems

Giulia Bonino*[1], Emanuele Di Lorenzo[3], Simona Masina[1], Doroteaciro Iovino[1]
[1] Euro-Mediterranean Center on Climate Change, Bologna, Italy
[2]Program in Ocean Science & Engineering, Georgia Institute of Technology, Atlanta, USA

Supplemental figures and tables

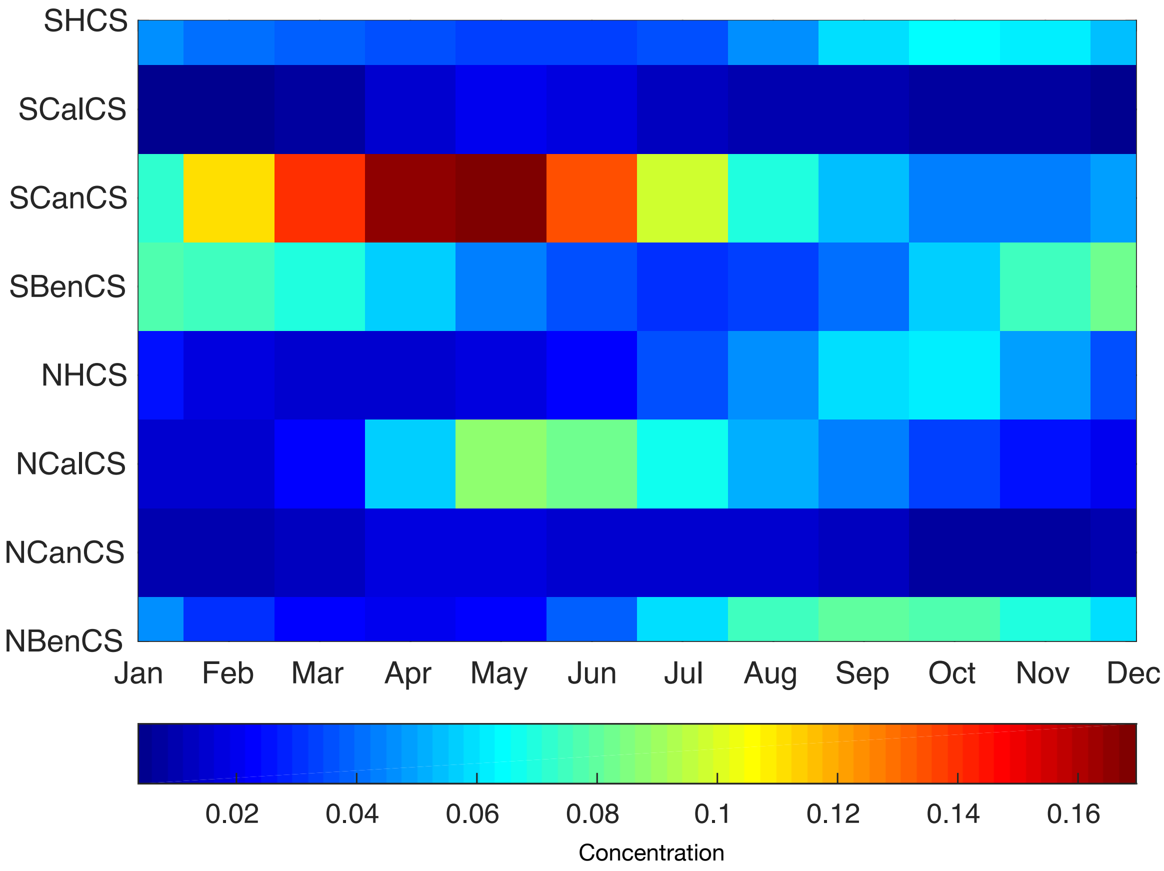

Supplementary Figure S1. *Seasonal cycle of subsurface tracers at surface. This figure was plotted using MATLAB R2017a (https://www.mathworks.com/products/new_products/release2017a.html).*

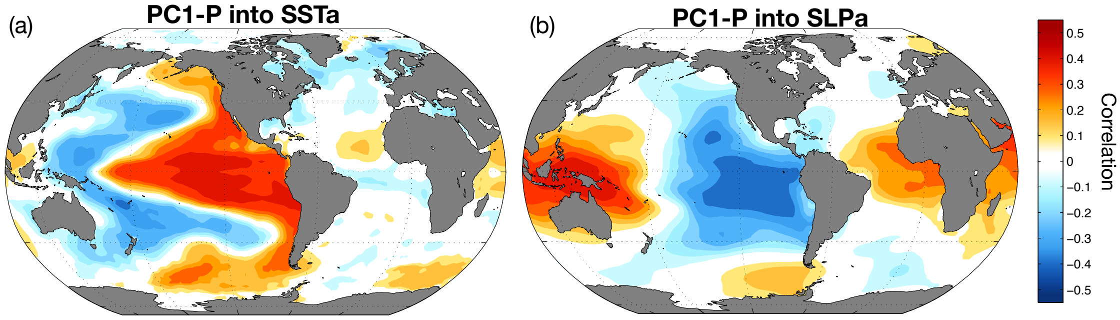

Supplementary Figure S2. *(a) Correlation patterns between PC1-P and SSTa; (b) Correlation patterns between PC1-P and SLPa. This figure was plotted using MATLAB R2017a (https://www.mathworks.com/products/new_products/release2017a.html).*
